# Melanoma Expressed-CD70 Is Regulated by RhoA and MAPK Pathways without Affecting Vemurafenib Treatment Activity

**DOI:** 10.1371/journal.pone.0148095

**Published:** 2016-02-01

**Authors:** Christine Pich, Iotefa Teiti, Guillaume Sarrabayrouse, Franck Gallardo, Rémi Gence, Anne-Françoise Tilkin-Mariamé

**Affiliations:** 1 INSERM UMR 1037, CRCT, Toulouse FR-31037, France; 2 Université Paul Sabatier, Toulouse FR-31062, France; 3 NeoVirTech, Institut des Sciences du vivant, Toulouse FR-31106, France; 4 INSERM U1220, IRSD, Toulouse FR-31024, France; University of Alabama at Birmingham, UNITED STATES

## Abstract

CD70 is a costimulatory molecule member of the Tumor Necrosis Factor family that is expressed on activated immune cells. Its ectopic expression has been described in several types of cancer cells including lymphomas, renal cell carcinomas and glioblastomas. We have recently described its expression in a part of tumor cells from the vast majority of melanoma biopsies and human melanoma cell lines, and found that CD70 expression decreased over time as the disease progressed. Here, we show that RhoA, BRAF and Mitogen Activating Protein Kinase pathways are involved in the positive transcriptional regulation of CD70 expression in melanomas. Interestingly, the clinical inhibitor of the common BRAF V600E/D variants, Vemurafenib (PLX-4032), which is currently used to treat melanoma patients with BRAF V600E/D-mutated metastatic melanomas, decreased CD70 expression in human CD70+ melanoma cell lines. This decrease was seen in melanoma cells both with and without the BRAFV600E/D mutation, although was less efficient in those lacking the mutation. But interestingly, by silencing CD70 in CD70+ melanoma cell lines we show that PLX-4032-induced melanoma cell killing and its inhibitory effect on MAPK pathway activation are unaffected by CD70 expression. Consequently, our work demonstrates that CD70 ectopic expression in melanomas is not a valuable biomarker to predict tumor cells sensitivity to BRAF V600 inhibitors.

## Introduction

Malignant melanomas are the most aggressive form of skin cancers that kills affected patients through multiple metastases [1]. Mortality rates increase in the advanced stages and patient survival following metastatic detection is usually short. Melanoma progression correlates with the appearance of molecular alterations, thereby generating more malignant tumors [2, 3]. Activating mutations in the serine/threonine kinase BRAF and in particular BRAFV600E/D mutations occur in about 50% of melanomas [1, 4]. These BRAF mutations induce activation of MAPK pathway, which is involved in essential cellular processes, such as proliferation, differentiation and particularly invasion suggesting a strong relationship between mutation and metastatic potential. PLX-4032 (also known as Vemurafenib) is a BRAF V600E/D specific inhibitor. Preclinical studies indicate that Vemurafenib blocks the mutated BRAF protein, triggering rapid cell growth arrest and cell death in tumors carrying these mutations [5]. Recent studies have shown that treatment with Vemurafenib also promotes anti melanoma immune response by enhancing tumor antigens expression, lymphocytes cytotoxicity and tumor infiltration by lymphocytes [6, 7]. Clinical trials of Vemurafenib have shown a therapeutic effect in more than 50% of patients with BRAF V600 mutated metastatic melanomas [5, 8]. Only patients with these mutations appear to benefit from the treatment, but for those patients Vemurafenib treatment has been shown to improve the rates of overall and progression-free survival and is recommended for the treatment of melanomas that have spread or cannot be removed by surgery. In the clinical context, the majority of patients first respond to this inhibitor and mostly, metastases uniformly regress. However, often cancer cells outbreak and progress again once resistance is acquired [7, 8].

Recently, using patients’ biopsies and human melanoma cell lines, we investigated the ectopic expression of CD70 in melanoma tumor cells [9]. CD70 is a costimulatory molecule and member of the TNF superfamily that is expressed in activated T- and B-lymphocytes. In these immune cells, CD70 is involved in priming, effector functions, differentiation, and memory formation through binding to its receptor, CD27 [10, 11]. The functional form of CD70 is a membrane-expressed homotrimeric type II molecule that, upon engagement, induces trimerization of the CD27 receptor to initiate intracellular signaling [10, 11]. CD70 also plays an intrinsic active role in T-lymphocyte activation. Indeed, cross-linking of CD70 with the CD70-specific mAb QA32 was shown to trigger T-cell mediated cytotoxicity, cytokine production, calcium mobilization and MAPK phosphorylation [12]. In agreement with this we have previously demonstrated that CD70-positive murine tumor cells co-expressing CD40L and H-2K(d) generated an enhanced anti-tumor immune response [13].

In addition to its expression in activated lymphocytes, CD70 expression has been documented in several types of lymphomas, glioblastomas [14] and renal cell carcinomas [15]. We recently showed that CD70 was expressed in most primary melanomas and that its expression was lost over the course of melanoma progression. This study also demonstrated that melanoma-expressed CD70 is implicated in tumor migration, invasion and metastasis. Melanoma cells expressing monomeric CD70 possessed reduced ability to migrate and invade surrounding areas, whereas the trimerization of CD70 increased the invasive potential of melanoma through MAPK pathway activation, RhoE overexpression and inhibition of actin fibers and focal adhesions [9].

Rho GTPases activity is central to melanoma cells, and indeed we have previously shown that RhoA inhibition induced the up-regulation of several immune-interacting molecules including MHC Class-I, CD80/CD86 costimulatory molecules and FasL, which like CD70 belongs to the TNF superfamily [16–18]. However, the correlation between Rho GTPases, BRAF and MAPK pathway activation status and CD70 expression in CD70+ melanoma cells is yet to be described.

Most Rho family members act as molecular switches, cycling between a GTP-bound active form and a GDP-bound inactive form. Once activated, Rho GTPases bind different effector molecules and trigger signaling cascades to direct essential cellular functions like cell growth, apoptosis and cytoskeletal dynamics involved in cell motility, invasion and metastasis [19]. The three highly-related Rho isoforms RhoA, B and C share some effector proteins, but show clear functional differences [20]. In this study, we investigated the roles of Rho GTPases on CD70 expression in human melanomas. In addition, we used BRAF specific siRNAs, PLX-4032 and several inhibitors of the MAPK pathway to investigate the role of this pathway in the regulation of CD70 expressed by melanoma cells. Our results showed that RhoA and MAPK pathway cross-talk to positively regulate melanoma-expressed CD70. Interestingly this CD70 expression does not affect PLX-4032-induced inhibition of MAPK pathway activation and tumor cell cytotoxicity.

## Materials and Methods

### Tumor cell lines

Human melanoma cell lines: LB1319-MEL (BRAF-wt and NRAS-wt) and LB39-MEL were kindly provided by Pr. T. Boon (Ludwig Institute for Cancer Research, Brussels). We have isolated a CD70+ clone of LB39-MEL cell line (LB39-MEL CD70+, BRAFV600E and NRAS-wt) by FACS sorting and cellular cloning. WM-266-4 (BRAFV600E/D and NRAS-wt) cell line was obtained from ATCC. These tumor cell line cultures were maintained by serial passages in RPMI 1640 medium (Lonza) supplemented with 10% FCS, 1 mM glutamine, and 1% penicillin-streptomycin-amphotericinB (Lonza). Cultures were tested monthly to ensure that they were mycoplasm-free.

### Treatments

Cells were directly treated with U0126 (Calbiochem) and PLX-4032 (Vemurafenib, Roche) at different concentrations for 24h (1,25 to 5 μM for U0126 and 0,25 to 1 μM for PLX-4032) to determine their specific effect on CD70 expression in cells.

Cells were first transfected with RhoA-siRNA, and after 48h treated U0126 5 μM for 24h.

### Flow cytometry analyses

PE-conjugated anti-CD70 mAb and isotype control were purchased from BD Biosciences. Stained cells were analyzed on a BD FACS Calibur (Becton Dickinson) and results were analyzed with FlowJo software. To evaluate CD70 membrane expression and to pool several analyses, the Fold Induction values (CD70 membrane expression FI corresponding to the normalized level of membrane-expressed CD70) were calculated compared to control conditions. In some experiments to evaluate membrane antigen expression on several independent experiments, the index of specific fluorescence (ISF) was determined. This was calculated using the following formula: (median fluorescence intensity (MFI) with the specific antibody—MFI with the isotype control) / MFI with the isotype control x 100.

### Western blot analyses

Cells were lysed in lysis buffer (20 mM Tris pH 7.6, 150 mM NaCl, 2 mM EDTA, 0.1% SDS, 0.5% NP-40, 1% proteases, and phosphatase inhibitor cocktail), and protein extracts were prepared by the standard procedure and then separated on sodium dodecyl sulfate–polyacrylamide (SDS-PAGE) gel electrophoresis (50 or 100 μg protein/lane). Proteins were blotted onto polyvinylidendifluoride membranes. The filters were incubated at 4°C overnight with primary antibodies against CD70, RhoA, RhoA-GTP, RhoB, RhoC, MEK1, P-MEK1, ERK1, ERK2, P-ERK1 and P-ERK2 (Santa Cruz Biotechnology and Cell Signaling). Actin was used as a loading control (Chemicon, Merck Millipore). The Hybond-p membranes (GE Healthcare) were then incubated with HRP-labeled secondary antibody (R&D System and Cell Signaling) for 1 h at room temperature and then detected with a chemiluminescence detection ECL kit (Thermo Scientific Pierce). Band intensities were quantified using ImageJ software (National Institute of Health, USA).

### Transfection of siRNAs and transduction with adenoviral vectors

Cells were transiently transfected with siRNAs, as previously described [16]. Briefly, melanoma cells (5 x 10^5^) were transfected with 20 nM siRNA using JetPrime (Polyplus Transfection). The following siRNA duplexes, purchased from Eurogentec, were used: siRhoA1 (GAAGUCAAGCAUUUCUGUC-TT); siRhoA2 (GCAGGUAGAGUUGGCUUUG-TT); siRhoB1 (CUAUGUGGGCCGACAUUGAG-TT); siRhoB2 (CCGUCUUCGAGAACUAUGU-TT); siRhoC1 (UAAGAAGGACCUGAGGCAA-TT); siRhoC2 (GACUAUGAUCGACUGCGGC-TT); siBRAF (GAGAAAUCUCGAUGGAGU-TT); a non-targeting siControl (siCtrl) (GACGUGGGACUGAAGGGGU-TT). The following siRNA duplexes, purchases from Thermo Fisher, were used: siCD70 pool (CACCAAGGUUGUACCAUUG, GCAUCUACAUGGUACACAU, GCAGCUGAAUCACACAGGA, UGACCACUGCUGCUGAUUA) and a non-targeting siNegative pool (siNeg) (UGGUUUACAUGUCGACUAA, UGGUUUACAUGUUGUGUGA, UGGUUUACAUGUUUUCUGA, UGGUUUACAUGUUUUCCUA).

Cells (6 x 10^5^) were transiently transduced with adenoviral vectors at a multiplicity of infection of 50:1 for 36h. Adenoviral vectors expressing RhoA (AdRhoA) under the transcriptional control of CMV promoter were constructed with the AdEasy System (MP Biomedical), according to the manufacturer’s instructions.

### Real-Time quantitative PCR (RT-qPCR)

Total RNA was isolated using the RNeasy kit (QIAGEN) according to the manufacturer’s instructions, then reverse-transcribed using the iScript cDNA synthesis kit (BioRad). Quantitative real-time PCR was performed with an iQreal-time PCR detection system (BioRad) using iQ SYBR Green Supermix (BioRad).

### Luciferase assays

The pCD70-FLuc plasmid reporting CD70 promoter activity with Firefly Luciferase (FLuc) was kindly given by Dr. B. Richardson (University of Michigan, Ann Arbor, MI) [21]. The pCMV-RLuc plasmid expressing Renilla Luciferase (RLuc) was co-transfected as an internal control. Cells were first transfected with siRhoA2 for 48 h, as described above. Cells were then plated in 24-well plates and transiently transfected with pCD70-FLuc and pCMV-RLuc plasmids using JetPEI (Invitrogen) according to the manufacturer’s instructions. Twenty-four hours after plasmid transfection, luciferase activities were measured using the Dual Luciferase Assay System (Promega).

### GST pull-down assay

The level of activated RhoA and GTP-bound RhoA protein was measured using the GST fusion protein containing the Rho binding domain of Rhotekin [22]. The amount of GTP- bound RhoA and the total amount of RhoA in cell lysates were determined by western blot as described above [23].

### Immunofluorescence

Untreated and treated cells were fixed and processed for immunofluorescence directed against CD70 using CytoFix/CytoPerm (BD Pharmingen) according manufacturers’ instructions, and stained for 45 min with PE-conjugated anti-CD70 mAb and isotype control (BD Pharmingen). CD70 expression based on immunofluorescence signal was quantified using Arrayscan VTi high content microscope (Thermo Scientific cellomics).

### In vitro proliferation

LB1319-MEL and WM-266-4 melanoma cells were transfected with siNeg or siCD70. Then cells were plated at 1x10^**4**^ cells and treated with 1 μM of PLX-4032. 72 h after treatment, cells were counted using Coulter Counter (Beckman Coulter).

### Statistical analysis

Statistical analyses were performed using GraphPad Prism software. Significance of RT-qPCR and CD70 promoter activity were assessed by t-test. Significance of flow cytometry and Western blot analyses were assessed by t-test or Tukey one-way ANOVA test. All statistic tests were two-sides. The values are expressed as means ± Standard Deviation (SD) in the figures. *P* values less than .05 were considered statistically significant.

## Results

### RhoA inhibition decreases CD70 expression in human melanoma cells

**Fig 1:**
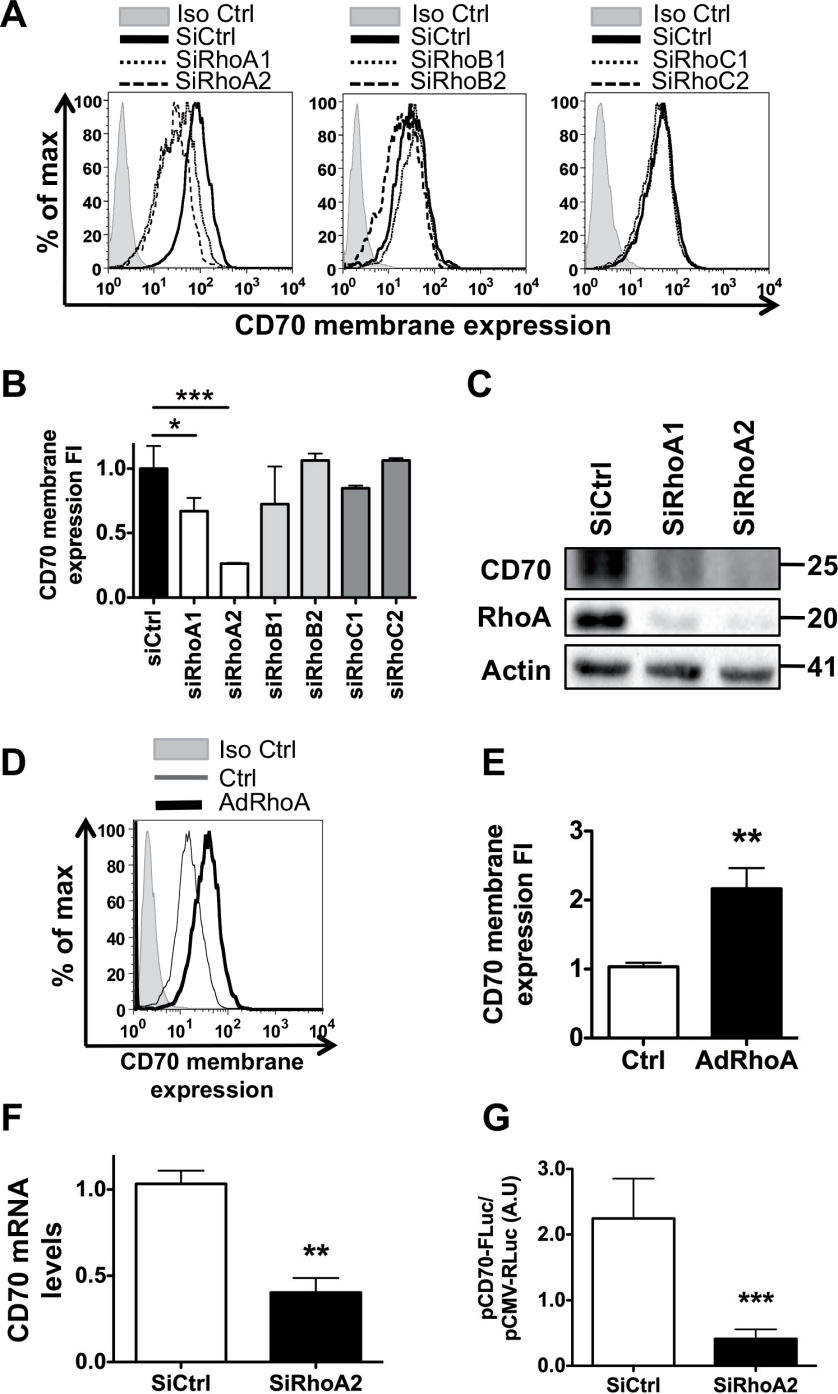
RhoA GTPase positively and transcriptionally controls CD70 membrane and global expression. LB1319-MEL cells were transfected with control siRNA (siCtrl), two RhoA-specific siRNAs (siRhoA1, siRhoA2), two RhoB-specific siRNAs (siRhoB1, siRhoB2), or two RhoC-specific siRNAs (siRhoC1, siRhoC2). 72 h post transfection, membrane associated CD70 levels were quantified using flow cytometry **(A)**. Quantification of three different experiments is shown in **(B)**. Western Blot analyses confirmed RhoA depletion and decreased in CD70 expression in LB1319-MEL cells 72 h post siRNA transfection. Actin was used as a loading control **(C).** RhoA over-expression was induced in LB1319-MEL cells by infection with an AdenoRhoA (AdRhoA). 36h post infection, levels of membrane associated CD70 were detected by flow cytometry **(D)**. Results of three different experiments are shown in **(E)**. siRhoA2 transfection in LB1319-MEL cells decreases the accumulation of CD70 mRNA, as detected by RT-qPCR **(F)**. Luciferase assay showed that downregulation of RhoA expression by siRhoA2 in LB1319-MEL cells represses CD70 promoter activity **(G)**. Flow cytometry histograms are illustrated in Fold induction (FI) corresponding to the normalized level of membrane expressed CD70. Results are expressed as mean values ± SD (error bars, *n* = 3 experiments). *P < 0.05; **P < 0.01; ***P < 0.001 versus control siRNA using the Tukey ANOVA test **(B)** or *t-test* **(E, G, H).**

As CD70 is not expressed in all melanoma cells [9] we choose three cell lines highly expressing CD70: LB1319-MEL (100% CD70+), LB39-MEL (CD70+ clones we isolated) and WM-266-4 (100% CD70+). These cell lines are also interesting for their different BRAF status, as LB1319-MEL is BRAF wt and LB39-MEL and WM-266-4 cells arbor BRAFV600E/D mutations. Since Rho GTPases are involved in tumor mobility and that we had previously shown that RhoA regulates the expression of another member of the TNF family (FasL) in melanoma cells [17], we tested for potential roles of Rho GTPases in the regulation of CD70 expression. We used a siRNA strategy to reduce RhoA, RhoB, or RhoC expression in CD70+ LB1319-MEL human melanoma cells. Transfections of two RhoA-specific siRNAs (siRhoA1 and siRhoA2) significantly reduced membrane-associated CD70, as detected by FACS analyses, compared to cells transfected with RhoB (siRhoB1 and siRhoB2), RhoC (siRhoC1 and siRhoC2), or control (siCtrl) siRNAs (Fig 1A and 1B). The efficiency of the siRNAs to decrease their specific Rho protein was controlled, as illustrated in Fig 1C and in S1A and S1B Fig. On the invert, infection of LB1319-MEL cells with RhoA-encoding adenoviruses induced RhoA overexpression and increased levels of membrane-associated CD70 (Fig 1D and 1E). Furthermore, transfection of LB1319-MEL cells with both RhoA-specific siRNAs significantly reduced global CD70 expression, as detected by Western blot (Fig 1C and S1C Fig). SiRhoA2 being the most efficient RhoA inhibitor, it was used in the subsequent experiments. Using, RT-qPCR, we show that CD70 mRNA accumulation is severely reduced after transfection with siRhoA2 compared to siCtrl (Fig 1F). To determine if this effect is linked to a modification of CD70 promoter activity, we used a plasmid expressing the firefly luciferase under the control of the CD70 promoter (pCD70FL reporter plasmid from [21]). We sequentially transfected siRhoA2 and pCD70FL plasmid, which induced a drastic decrease of Luc signal showing that RhoA positively regulated CD70 promoter (Fig 1G).

To confirm RhoA involvement in the regulation of melanoma-expressed CD70, two others human CD70+ melanoma models: WM-266-4 cells and CD70+ clone of LB39-MEL cell line were used. Membrane associated and total CD70 protein levels were decreased in these cells after RhoA specific siRNA transfections (S1D–S1G Fig).

Altogether, these data show that RhoA positively controls transcriptional levels of ectopic CD70 in human melanoma cells.

### Inhibition of the MAPK pathway decreases CD70 expression

**Fig 2:**
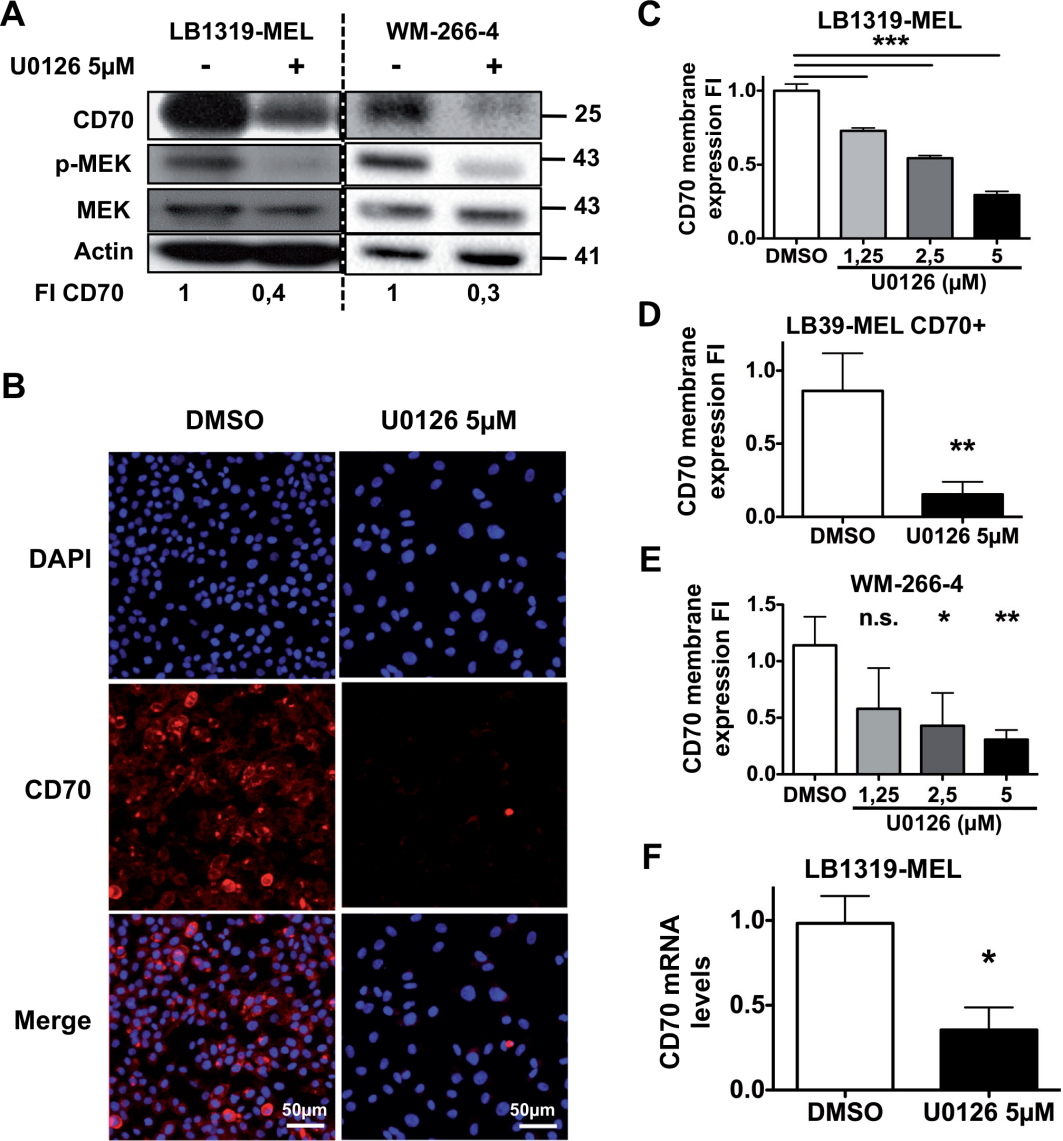
MEK kinase positively and transcriptionally controls CD70 membrane and global expression. LB1319-MEL and WM-266-4 cells were treated with 5 μM of U0126 for 72 h then analyzed by Western Blot for CD70, phospho-MEK and MEK expression. Actin was used as a loading control **(A)**. LB39-MEL CD70+ cells were treated with 5 μM of U0126 for 72 h then fixed and processed for immunofluorescence directed against CD70. Pictures of U0126 treated cells and control (DMSO) conditions are presented (scale bar 50 μm) **(B)**. LB1319-MEL cells were treated or not with U0126 for 72 h at indicated concentrations then analyzed by flow cytometry for membrane CD70 expression. Fold induction of CD70 membrane expression in triplicate condition is illustrated **(C)**. Same experiments were performed in LB39-MEL CD70+ cells **(D)** and WM-226-4 cells **(E)**. In LB1319-MEL cells treatment with 5 μM of U0126 for 72 h decreases the accumulation of CD70 mRNA, as detected by RT-qPCR **(F)**. Results are expressed as mean values ± SD (error bars, *n* = 3 experiments). *P < 0.05; **P < 0.01; ***P < 0.001 versus control siRNA using the Tukey ANOVA test **(C, E)** or t-test **(D, F)**.

We next tested the implication of the MAPK pathway in the regulation of melanoma- expressed CD70, because this pathway is involved in TNF family protein regulation [21] and is activated in many melanomas, where it plays an essential role in melanoma progression and metastasis [4, 5]. Constitutive MAPK pathway activation was observed in the three CD70+ cell lines tested (LB1319-MEL, LB39-MEL CD70+ and WM-266-4), as phosphorylated MEK and ERK were detected by Western blot without any stimulation (S2A Fig). To test MAPK pathway implication in the regulation of melanoma-expressed CD70, we used two specific pharmacological inhibitors of MEK phosphorylation (p-MEK): PD98059 (data not shown) and U0126. Melanoma cells were treated for 48 h at different concentrations. Inhibition of MAPK pathway activation was observed, as illustrated in LB1319-MEL and WM-266-4 cells, by reduction of p-MEK (Fig 2A). Down-regulation of membrane-expressed and global CD70 proteins was observed in cells treated with these two inhibitors. Fig 2B illustrates this decrease in CD70 expression in LB39-MEL CD70+ cells treated with U0126 at 5μM. Enlarged nuclei in the U0126 condition is linked to MEK inhibition, as treatment with another MEK inhibitor (PD184161), strongly decreasing CD70 accumulation, triggers the same nuclei enlargement (data not shown). Histograms in Fig 2C, 2D and 2E demonstrate the U0126 dependent decrease of membrane-expressed CD70 in LB1319-MEL, LB39-MEL CD70+ and WM-266-4 cells respectively. U0126 inhibitor was less toxic than PD98059, so it was used for the following experiments. Total amount of CD70 proteins, detected by Western blot (Fig 2A and S2B Fig), and CD70 mRNA level, detected by RT qPCR (Fig 2F) were also reduced by the treatment with U0126 inhibitor at 5μM.

These results demonstrate that MEK protein activation is involved in the positive and transcriptional regulation of CD70 expression in melanoma cells.

### BRAF silencing and treatment of melanoma cells with PLX-4032 reduce CD70 expression

In most of cases in melanomas, MAPK pathway constitutive activation is associated with BRAF activated mutation. Vemurafenib (PLX-4032) is a pharmacological molecule designed to inhibit the constitutive activation of the BRAFV600 mutated proteins involved in melanoma metastasis [5].

**Fig 3:**
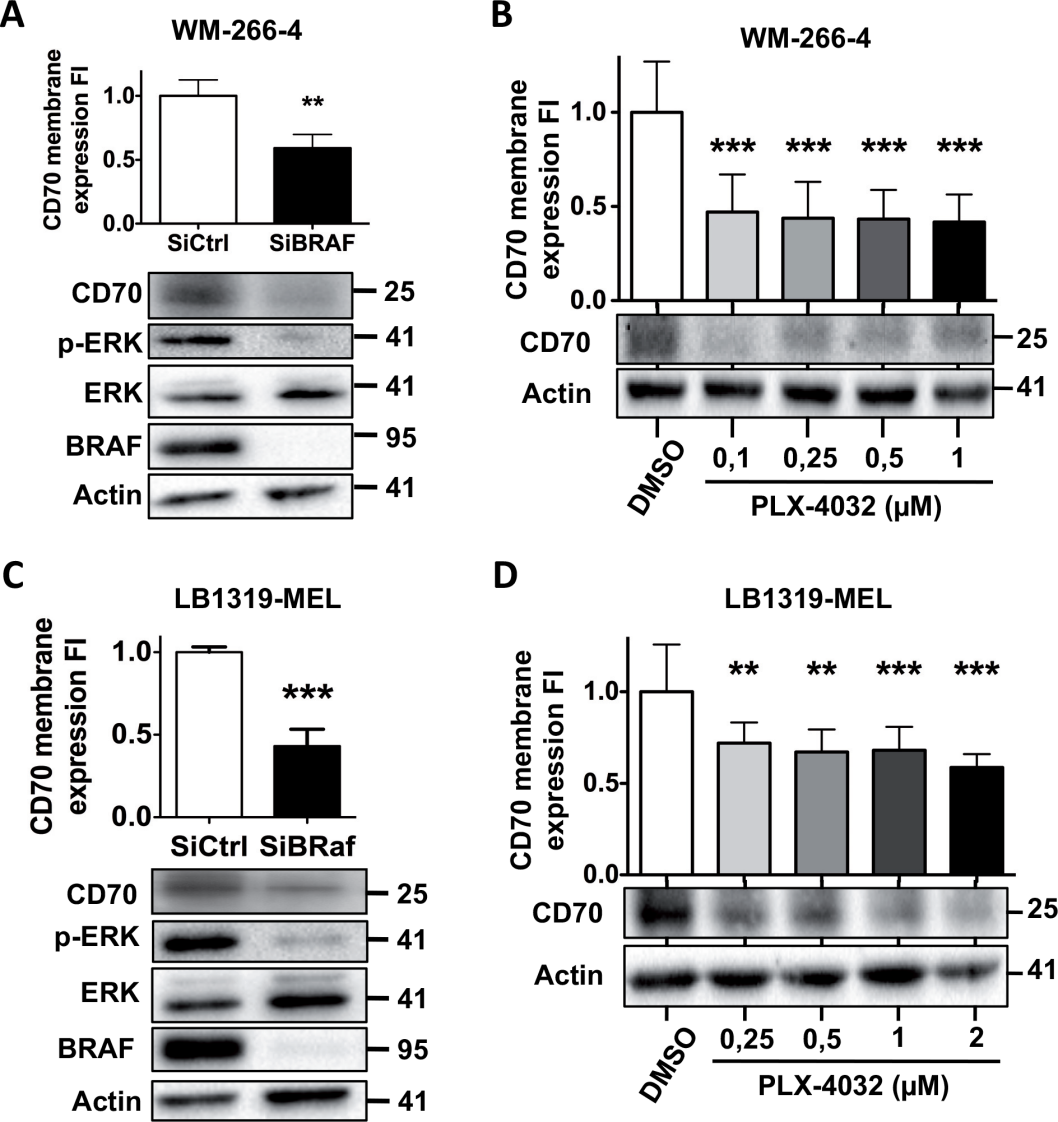
BRAF protein positively controls CD70 membrane and global expression. WM-266-4 cells were transfected with control siRNA (siCtrl) and BRAF-specific siRNA (siBRAF). Cells were analyzed after 72 h of transfection by flow cytometry for membrane CD70 expression **(A-upper)** and by Western Blot for BRAF, global CD70, phospho-ERK and total ERK expression. Actin was used as a loading control **(A-lower)**. Same experiments have been performed in LB1319-MEL cells **(C)**. WM-266-4 cells were treated with control medium (DMSO) or with PLX-4032 at indicated concentrations for 72 h then analyzed by flow cytometry for CD70 membrane expression **(B-upper)** or by Western Blot for CD70 global expression. Actin was used as a loading control **(B-lower)**. Same experiments have been performed in LB1319-MEL cells **(C)**. Cytometry results are expressed as mean values ± SD (error bars, *n* = 3 experiments). **P < 0.01; ***P < 0.001 versus control condition (DMSO or siCtrl) using the *t-test* **(A, C)** *or* Tukey ANOVA test (**B**, **D**). Western Blot illustrations are representative of three independent experiments.

MAPK pathway activation observed in LB39-MEL CD70+ and WM-266-4 cells is linked to BRAFV600E mutation, but in LB1319-MEL cells this activation is not due to BRAFV600 mutation, because these cells are not mutated at this locus (S2 Fig). Silencing BRAF in these melanomas inhibited MAPK pathway activation, which is illustrated by the reduction of ERK phosphorylation (Fig 3A and 3C), so we tested the involvement of BRAF protein in melanoma-expressed CD70 using a BRAF specific siRNA. This transfection also reduced membrane and total CD70 compared to cells transfected with the control siRNA (Fig 3A and 3C). Then the pharmacological inhibitor of BRAFV600 mutation: PLX-4032 was tested. *In vitro* treatment of the three CD70+ melanoma cell lines with PLX-4032 induced membrane and global CD70 decrease (Fig 3B–3D and S3A–S3D Fig). This effect was observed in the two-melanoma cell lines bearing the mutation V600E (LB39-MEL CD70+ and WM-266-4) (Fig 3B and S3A–S3C Fig) and unexpectedly also in LB1319-MEL cells, which do not have BRAF V600 mutation. However in LB1319-MEL cells the effect was lower and obtained with higher doses of PLX-4032 (Fig 3D and S3D Fig).

These results demonstrated that BRAF regulate CD70 expression independently of the V600E mutation and confirm that MAPK pathway activation positively regulates melanoma-expressed CD70 and that inhibition of this pathway at BRAF or MEK level are both able to reduce melanoma expressed CD70.

### RhoA and MAPK pathways are associated to regulate melanoma-expressed CD70

**Fig 4:**
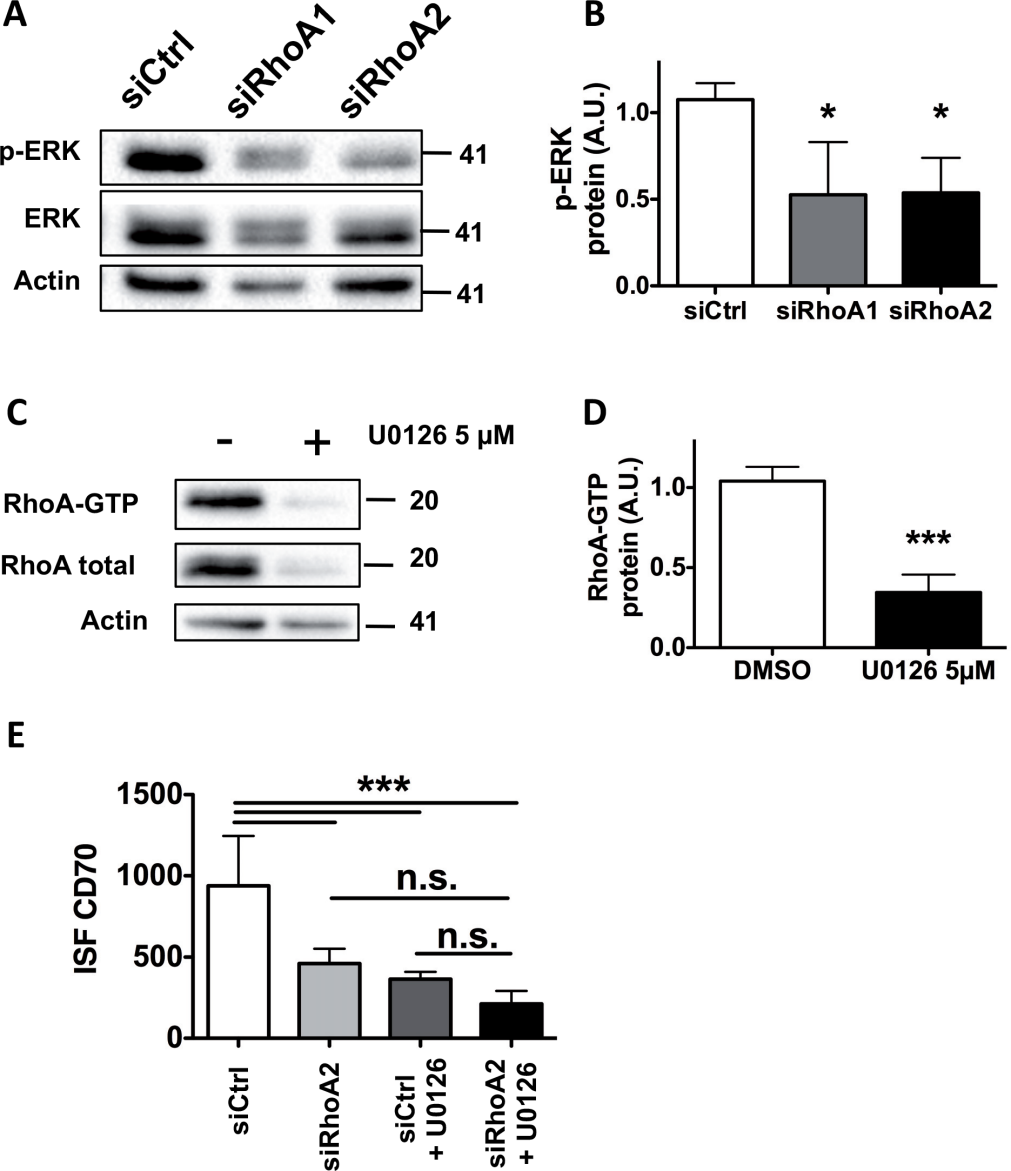
RhoA and MAPK pathways are associated to regulate C70 expression. LB1319-MEL cells were transfected with control siRNA (siCtrl) and two RhoA-specific siRNAs (siRhoA1, siRhoA2). Western Blot analyses were performed 72 h after transfection for phospho- and total-ERK expression. Actin was used as a loading control **(A)**. Quantification of three independent experiments is shown in **(B)**. LB1319-MEL cells were treated with 5μM of U0126 for 72 h then analyzed by the TRBD pull-down assay (Rho binding domain of Rhotekin) **(C)**. Quantification of three independent experiments is shown in **(D)**. LB1319-MEL cells were transfected with control siRNA (siCtrl) or siRhoA2 for 72 h. At the same time (24 h after transfection), the same cells were treated or not with 5μM of U0126 for 48 h. Finally, cells were analyzed by flow cytometry for CD70 membrane expression. Quantification of three independent experiments by ISF is shown in **(E)**. Results are expressed as mean values ± SD (error bars, *n* = 3 experiments). *P < 0.05; ***P < 0.001 versus control (siCtrl or DMSO) using the Tukey ANOVA test **(B, E)** or t-test **(D)**.

Both RhoA and MAPK are critical components of cellular signal transduction pathways. Hyperactivity and over-expression of RhoA and hyper-activated mutated BRAF have been observed in human cancers, including melanoma [8, 20]. Our data, as previously described in melanoma cells [24], show that proteins of the MAPK pathway cross-talk with RhoA. Here we show that this cross-talk is responsible for the regulation of CD70 expression. MAPK pathway activation was evaluated after RhoA inhibition induced by transfection with specific siRNAs (siRhoA1 and siRhoA2) in LB1319-MEL cells, and conversely RhoA expression and activation were evaluated after treatment with the MEK phosphorylation inhibitor: U0126. The results, illustrated in Fig 4A–4D, show that these two pathways are linked. Indeed, silencing RhoA decreased ERK phosphorylation (Fig 4A and 4B), and conversely inhibition of MAPK pathway using U0126 inhibitor induced decreased expression and activation of RhoA (Fig 4C and 4D). Moreover, LB1319-MEL cells were simultaneously transfected with siRhoA2 versus siCtrl and treated by U0126 at 5μM. The observed membrane-expressed CD70 down-regulations induced by siRhoA2 transfection or U0126 treatment were not increased by the combination of both treatments (Fig 4E), indicating that RhoA and MAPK pathway have no additive effects on the regulation of melanoma-expressed CD70.

Altogether these results demonstrated that RhoA and MAPK pathways are associated in positive and transcriptional regulation of CD70 expression in melanoma.

### CD70 expression in melanomas does not interfere with PLX-4032 inhibitory activity on MAPK pathway activation and tumor cells proliferation

**Fig 5:**
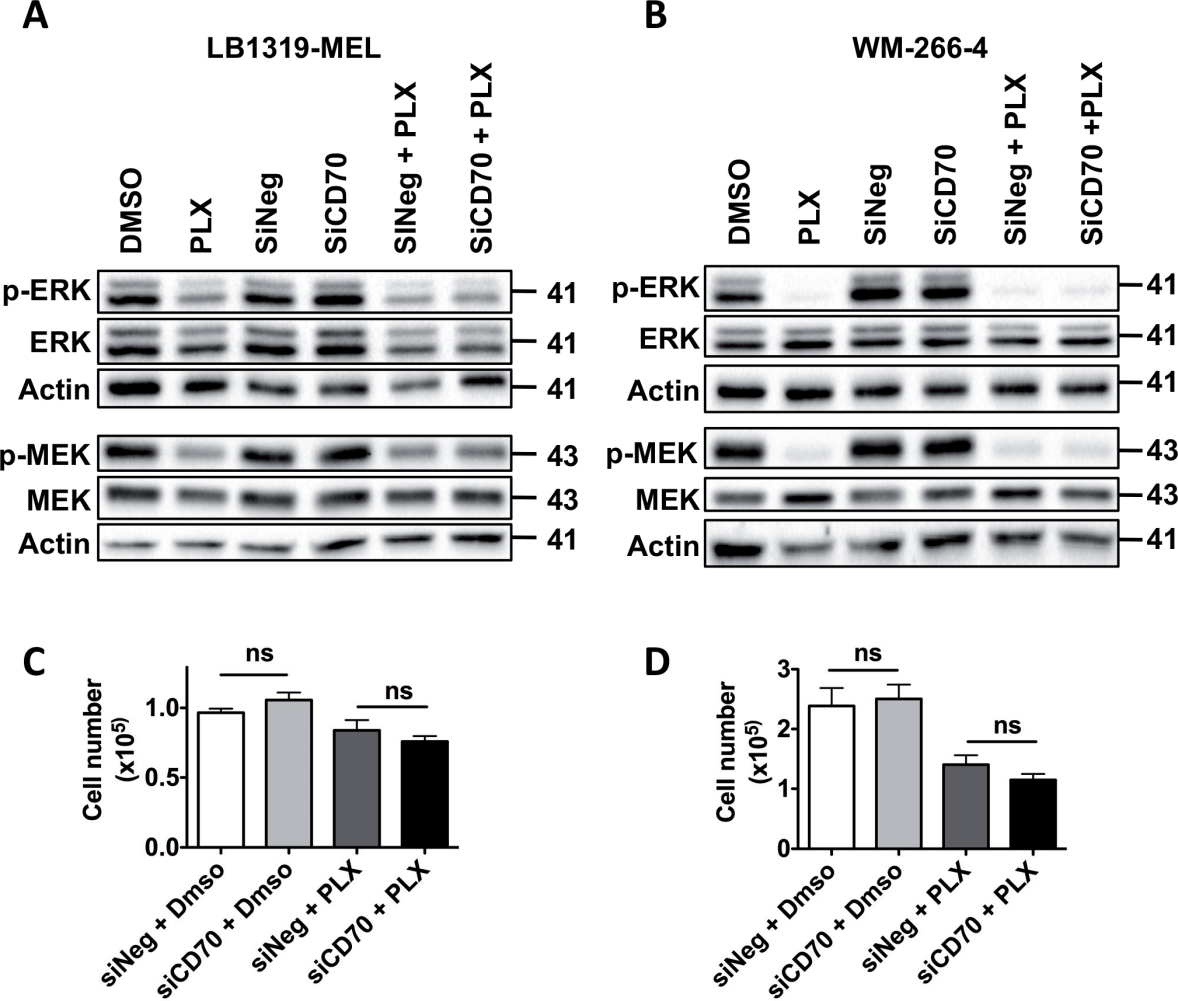
CD70 expression does not interfere with PLX-4032-induced inhibition of MAPK pathway and tumor cells killing. LB1319-MEL cells were transfected with control siRNA (siCtrl) or a CD70-specific siRNA (siCD70) for 72 h. At the same time, these cells were treated or not with 1μM of PLX-4032 (PLX) for 72 h. Then cells were analyzed by Western Blot for phospho- and total-ERK expression and phospho- and total-MEK expression. Actin was used as a loading control **(A)**. Same experiments have been performed in WM-266-4 cells **(B)**. Illustrations are representative of three independent experiments. Western Blot illustrations are representative of three different experiments. LB1319-MEL (**C**) and WM-266-4 (**D**) melanoma cells were transfected with siNeg or siCD70. Then cells were plated at 1x10^**4**^ cells and treated with 1 μM of PLX-4032. 72 h after treatment, cells were counted using Coulter Counter. Quantification of three independent experiments is shown as mean values ± SD. Non-significant (ns) *p*-value using the Tukey ANOVA test (**C**, **D**).

We wondered if CD70 expression in melanomas might interfere with PLX-4032-induced inhibition of MAPK pathway activation and PLX-4032-induced melanoma killing. To test this hypothesis, we evaluated MAPK pathway activation in CD70+ melanoma cells (LB1319-MEL and WM-266-4) after simultaneous silencing of CD70 and treatment with PLX-4032. Specific silencing of CD70 by siCD70 was confirmed in LB1319-MEL and WM-266-4 cells (S4A and S4B Fig). The observed down-regulation of MAPK pathway activation induced by PLX-4032 was not altered in cells transfected by siCD70 compared to siNeg (Fig 5A and 5B). Moreover, simultaneous silencing of CD70 and treatment with PLX-4032 did not alter PLX-4032-induced inhibition of tumor cells proliferation (Fig 5C and 5D). Altogether these results showed that melanoma-expressed CD70 does not interfere with PLX-4032 effects, suggesting that CD70 is not a biomarker of melanoma sensitivity to Vemurafenib (PLX-4032) treatment.

## Discussion

Altogether our experiments show that the expression of CD70 in melanoma cells is under the control of RhoA and the MAPK pathway. Most primitive melanomas contain CD70-positive tumor cells, and this expression decreases during the progression of the disease and in metastasis. In melanoma cell lines the monomeric form of CD70 is associated with reduced migration, invasion and metastasis capacities, whereas the active trimeric form of CD70 induces the activation of a signaling pathway, which favors melanoma invasiveness [9]. Therefore it is not surprising that CD70 expression is tightly regulated in these tumor cells.

In melanomas, like for CD70, the expression of 1α-Hydroxylase (CYP27B1), the enzyme responsible for the synthesis of vitamin D, decreases during the progression of the disease. The reduction of CYP27B1 correlates with melanoma phenotype and behaviour indicating a role in the pathogenesis and progression of this cancer. Moreover CYP27B1 is inversely related to melanin expression [25,26]. Despite this similitude in the evolution of CD70 and CYP27B1 expression during melanoma progression, we have observed that the expression of CD70 and melanin are not correlated in the melanoma cell lines used in this study. Indeed LB1319-MEL cells are CD70 and melanin positive and WM-266-4 are CD70 positive and they express low a level of melanin, and among our CD70 negative melanoma cell lines certain are melanin positive and other are negative.

Using a human radial growth phase melanoma cell line (WM35), Ruth *et al*. previously showed that over-expression of RhoC promoted cell invasion by two independent pathways involving either ROCK or PI3K/Akt [27]. Here, using three other human melanoma cell lines we have shown that RhoA, rather than RhoC, controls the migration and invasive capacities of melanoma cells through its positive regulation of melanoma-expressed CD70. RhoA is a key player in many different types of cancer cells. Indeed, several different tumor models, including melanomas, have been used to implicate the RhoA GTPase in tumor growth, cell motility and metastatic development [19, 28, 29].

The role of the BRAF/ERK pathway on CD70 expression in melanoma cells was also investigated here because this pathway is essential in melanoma progression and metastasis [4], and because pharmacological inhibitors have a strong but often transitory efficacy against these tumors. The interactions between the MAPK pathway and CD70 are complex and form a loop of positive regulation. Indeed, we have demonstrated here that the MAPK pathway positively regulates CD70 expression in melanoma cells. We have previously shown that the enhancement of the active trimeric form of CD70, facilitated by the binding of the CD70-specific mAb QA32, induced MAPK pathway hyper-activation, which favored melanoma cell invasion [9]. Moreover, anti-CD70 antibodies are considered as promising antibodies to treat human malignancies [30], inducing apoptosis via CD27 interaction and used to deliver drugs into the tumor. However, they were not tested in melanoma. These results revealed a role for CD70 in melanoma invasiveness via its interaction with MAPK pathway activation and suggested that anti-CD70 antibodies should be selected to eliminate those that induce CD70 trimerization and consequently promote tumor invasion.

Linkage between the RhoA-ROCK and the BRAF-ERK-MAPK pathway has previously been described, for example in the control of osteogenic gene expression [31] and in colon adenocarcinoma cells, where it induced cell migration and invasion [32]. Here we have shown that RhoA and MAPK pathway cross-talk to regulate melanoma-expressed CD70. But we have also shown that the interaction of melanoma-expressed CD70 with the MAPK pathway does not interfere with the inhibitory activities of PLX-4032 (Vemurafenib) in melanoma cells on MAPK pathway activation and melanoma cell survival. This information is essential for the clinical use of Vemurafenib, to know that presence or absence of CD70 in melanoma patient tumor cells will not affect the efficacy of Vemurafenib treatment. Consequently patients' biopsies do not need to be tested for CD70-melanoma expression before treatment of patients with Vemurafenib treatment and it does not interfere with the treatment efficacy.

## Supporting Information

S1 Fig RhoB (Figure A) et RhoC (Figure B) silencing were checked by Western Blot in LB1319-MEL cells transfected with siCtrl, siRhoB1, siRhoB2, siRhoC1 or siRhoC2 for 72 h. LB1319-MEL cells were transfected with siCtrl, siRhoA1 and siRhoA2. 72 h post transfection CD70 global expression was analyzed by Western Blot and quantified in three independent experiments in (Figure C). LB39-MEL CD70+ cells were transfected with control siRNA (siCtrl) and siRhoA2. 72 h post transfection membrane associated CD70 was quantified using flow cytometry. Quantification of three different experiments is shown in (Figure D). WM-266-4 cells were also transfected with siCtrl, siRhoA1 and siRhoA2. 72 h post transfection membrane associated CD70 was quantified using flow cytometry. Quantification of three different experiments is shown in (Figure E). In the same cells CD70 global expression was also analyzed by Western Blot as illustrated in (Figure F) and quantified in three independent experiments in (Figure G). Results are expressed as mean values ± SD (error bars, *n* = 3 experiments). *P < 0.05; **P < 0.01; ***P < 0.001 versus control condition (siCtrl) using the t-test (Figure D) or the Tukey ANOVA test (Figures C, E, G).
(EPS)

S2 Fig MAPK pathway activation was evaluated by Western Blot in LB1319-MEL, LB39-MEL CD70+ and WM-266-4 cells (Figure A). WM-266-4 cells were treated with 5μM of U0126 for 48 h and analyzed for CD70 global expression by Western Blot. Quantification of three independent experiments is shown in (Figure B). Results are expressed as mean values ± SD (error bars, *n* = 3 experiments). ***P < 0.001 versus control condition (DMSO) using the t-test **(Figure B).**
(EPS)

S3 Fig LB39-MEL CD70+ cells were treated with control medium (DMSO) or different doses of PLX-4032 for 72 h, and analyzed by flow cytometry for CD70 membrane expression (Figure A) or by Western Blot for CD70 global expression (Figure B). Quantification of three independent experiments is shown in Figure B-upper and a representative Western blot illustration is shown in Figure B-lower. Actin was used as a loading control. Same experiments were done for WM-266-4 and LB1319-MEL cells lines. Quantification of CD70 global expression by Western Blot is shown in (Figure C) for WM-266-4 cells and in (Figure D) for LB1319-MEL cells. Results are expressed as mean values ± SD (error bars, *n* = 3 experiments). *P < 0.05; **P < 0.01; ***P < 0.001 versus control condition (DMSO) using the Tukey ANOVA test **(Figures A, B, C, D).**
(EPS)

S4 Fig CD70 silencing were checked by Western-Blot in LB1319-MEL cells (Figure A) and in WM-266-4 (Figure B) transfected with control siRNA (siNeg) or CD70 specific siRNA (siCD70). 72 h post transfection CD70 global expression was analyzed by Western-Blot **(Figures A, B)**.
(EPS)
